# A novel home-based rehabilitative knee brace system is a viable option for postoperative rehabilitation after anterior cruciate ligament reconstruction: a report of 15 cases

**DOI:** 10.1186/s40634-022-00538-z

**Published:** 2022-09-23

**Authors:** Chih-Kai Hong, Zhao-Wei Liu, Kai-Lan Hsu, Fa-Chuan Kuan, Jeng-Feng Yang, Wei-Ren Su

**Affiliations:** 1 Department of Orthopaedic Surgery, National Cheng Kung University Hospital, College of Medicine, National Cheng Kung University, No.138, Sheng-Li Road, Tainan City, 70428 Taiwan; 2 Skeleton Materials and Bio-Compatibility Core Lab, Research Center of Clinical Medicine, National Cheng Kung University Hospital, College of Medicine, National Cheng Kung University, Tainan, Taiwan; 3 Physical Therapy Center, National Cheng Kung University Hospital, College of Medicine, National Cheng Kung University, Tainan, Taiwan; 4 Department of Physical Therapy, College of Medicine, National Cheng Kung University, Tainan, Taiwan; 5 Musculoskeletal Research Center, Innovation Headquarter, National Cheng Kung University, Tainan, Taiwan

**Keywords:** Telehealth, Telerehabilitation, Home-based rehabilitation, Anterior cruciate ligament, Knee brace

## Abstract

**Purpose:**

To investigate the functional outcomes for patients who used a novel home-based rehabilitative system during the postoperative period after anterior cruciate ligament (ACL) reconstructions.

**Methods:**

Patients undergoing ACL reconstruction surgeries were prospectively enrolled. A home-based rehabilitation system, which is composed of a knee brace with a motion tracker, a mobile app, and a web portal, was applied. Patients could complete the rehabilitation exercise through the audio guidance and the real-time tracking system which displayed the achieved motions on the user interface of the app. Feedbacks from the patients, including the International Knee Documentation Committee (IKDC) scores, were collected and uploaded to the web portal. Each patient would meet a specialized physical therapist face-to-face once a month. At postoperative 6 months, every patient received a GNRB arthrometer examination and a Cybex isokinetic dynamometer examination.

**Results:**

A total of 15 patients (10 males and 5 females) were enrolled and followed for at least 6 months. The mean time of return to full knee extension was 1.5 months.

The mean difference in laxity measured by GNRB arthrometer at 134 N significantly improved at postoperative 6 months (1.8 ± 1.6 mm) compared to that measured preoperatively (3.4 ± 1.9 mm) (*p* = 0.024). The peak torques of flexor and extensor muscles measured by Cybex isokinetic dynamometer remained unchanged at postoperative 6 months (*p* = 0.733 and 0.394, respectively). The patients’ IKDC score became smaller at postoperative 1 month (*p* = 0.011) and significantly improved at postoperative 6 months (*p* = 0.002).

**Conclusion:**

Using a home-based rehabilitative knee brace system after ACL reconstruction is a viable option as patients maintained their knee muscle strengths maintained their muscle strength and achieve similar or better knee range of motion six months postoperatively.

## Introduction

Anterior cruciate ligament (ACL) injury is one of the most common athletic knee injuries. In addition to the surgery itself, rehabilitation is the key to a successful outcome after ACL reconstruction [4]. Not only the biologic healing timelines of tendon grafts but also the successful completion of criterion-based milestones is critical for proving return to sport decision-making for the patients [4].

Digital health technology for rehabilitation has been considered an effective, low-cost, and accessible option to help patients resume physical function after surgery [17]. Telehealth is also an attractive option for health care during the COVID-19 outbreak as it reduces the personal contacts and overcomes travel restrictions [13]. It is suggested that telehealth physical therapy is non-inferior to conventional face-to-face physical therapy for several musculoskeletal disorders and selected suitable patients [9, 15].

As the telerehabilitation is developing, the present study aimed to investigate the functional outcomes for patients who used a novel home-based rehabilitative system, which is composed of a knee brace with a motion tracker, a mobile app, and a web portal, during the postoperative period after anterior cruciate ligament (ACL) reconstructions. The clinical criterion-based milestones were set up in this rehabilitation system [3]. We hypothesized that patients who received the home-based rehabilitative knee brace system have acceptable functional outcomes and knee stability in the postoperative 6 months.

## Methods

### Study design and population

The study was a prospective case series. This study was approved by the Institutional Review Board of National Cheng Kung University Hospital, Tainan, Taiwan (ID No. A-ER-l09-121). Written informed consent was obtained from each participant in this study. Patients aged 18–50 years with ACL complete ruptures with/without meniscus tears and who planned to undergo ACL reconstruction surgeries were prospectively enrolled. Patients who refused the invitation, had concomitant knee ligament injuries, had dermatological problems affecting the thigh and leg, had other unstable lower-extremity orthopedic conditions, or did not have suitable electronic devices for installing apps were excluded. The patient-enrollment period was from May 1, 2020, to April 30, 2021, with follow-up through October 31^st^, 2021. Each patient was followed for at least 6 months.

### KNEESUP Compact home-based rehabilitation system

**Fig. 1:**
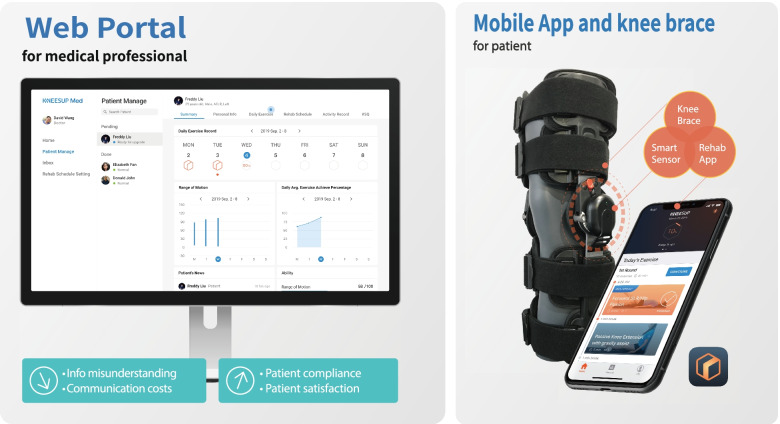
KNEESUP Compact home-based rehabilitation system. It is composed of a smart core motion tracker, a mobile App and a web portal

KNEESUP Compact (Conzian Ltd., Taipei, Taiwan) home-based rehabilitation is composed of a smart core motion tracker, a mobile App and a web portal (Fig. 1). The KNEESUP care App can be installed in mobile devices and be connected to a motion tracker, called smart core, on the knee brace via Bluetooth. Patients’ rehabilitation status, including the completion of daily schedule and feedback to their healthy status, were stored on the web server through the internet. Orthopedic doctors and physical therapists could then follow up the status of each patient by using electronic devices that could access to the website.

#### Motion tracker – smart core

A smart core motion tracker includes accelerometers, angle sensors and gyroscopes that can track motions in three-dimensional space. The accuracy of the motion tracker has been verified by Industrial Technology Research Institute, Taiwan. The smart core motion tracker communicates with mobile devices via Bluetooth 4.2 with the 10 Hz sampling frequency. As shown in Fig. 1, the single smart core is mounted on a knee brace to detect lower limb motions.

#### Mobile app – KNEESUP care

**Fig. 2:**
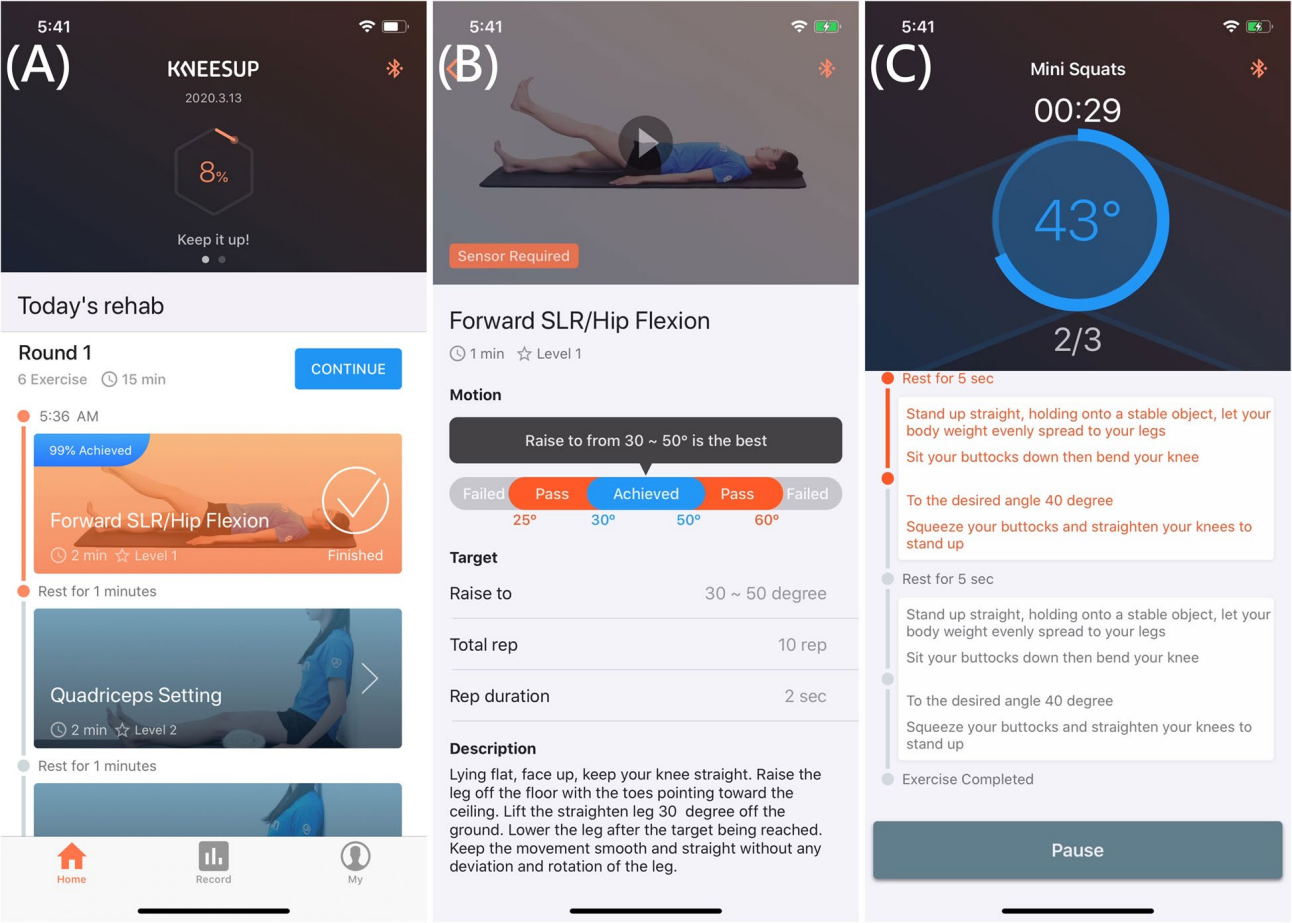
**A** The home page including rehabilitation plan, **B** the instructional page, and **C** the rehabilitation execution page on KNEESUP Care App

To carry out home rehabilitation, patients are asked to install KNEESUP Care app, which is available on both Google Play for android devices and Apple Store for IOS devices. Once a patient is registered and connected to a registered clinical staff (either an orthopedic doctor or a physical therapist), a tailored rehabilitation schedule will be sent to the app (Fig. 2A). To prevent misunderstanding of the rehabilitation exercise, a demonstration video with explanation of the exercise is illustrated to the patients when it was executed for the first time (Fig. 2B). Patients can execute the rehabilitation exercise not only through the audio guidance but also through the real-time tracking system that displayed the achieved motions on the user interface of the app (Fig. 2C). After finishing all target exercises, the KNEESUP Care app will upload the data collected by the smart core sensor to the internet server. Feedbacks from the patients after each exercise program are also collected and uploaded to the web portal. Patients can check their daily exercise reports or watch the exercise demonstration video through the user interface anytime.

#### Web portal – KNEESUP Med

Orthopedic doctors or physical therapists can design the rehabilitation schedules and monitor the daily status of each patient through this web portal. KNEESUP Med provides a friendly user interface that illustrates the unfinished tasks of the patients and negative feedbacks from the patient, such as pain and any adverse events, in the home page. Medical staff can set up a new rehabilitation plan or modify the existing rehabilitation plans for patients in this web portal. The raw data collected from the smart core will be found in this web portal, and the summary of the daily exercise is also illustrated. Medical staffs can adjust the rehabilitation schedules or replace the existing plans for the patient timely based on the daily reports from the patients.

### Preoperative managements

All patients completed pre-operative magnetic resonance imaging (MRI) evaluations. A specialized physical therapist (Z-W L) met each patient and explain both the preoperative and postoperative rehabilitation programs to them. The KNEESUP Compact home-based rehabilitation system was introduced to the included patients but was used in postoperative period only. Patients were asked to install KNEESUP Care app in their mobile devices. An instructional lecture on the utilization of KNEESUP Compact rehabilitation system was given to the patients. All patients completed both a GNRB arthrometer examination (Genourob, Laval, France) and a Cybex isokinetic dynamometer examination (CYBEX, MA, USA) preoperatively. The thigh circumference.

The thigh circumference was measured 15 cm proximal to the superior pole of the patella, which was in accordance with the previous study [19].

### Surgical technique

The arthroscopy ACL reconstruction was performed using a single bundle reconstruction technique using hamstrings tendon autograft by a single senior arthroscopic surgeon (W-R S.). Concomitant meniscus tears, if any, were repaired whenever possible during the primary ACL reconstruction. The reconstructed tendon graft was fixed with a suspensory button device (Endobutton, Smith & Nephew Inc., MA, USA) on the femur side, whereas it was finally fixed with a bio-interference screws and was fixed to a post screw using 6.5 mm cancellous screw with washer on the tibial side.

### Postoperative managements

The knee brace of the KNEESUP Compact system was applied to the affected knee immediately after the surgery. The patients followed the rehabilitation exercise programs in the app. The default rehabilitation programs and criterion-based milestones were summarized in Table 1. Individualized adjustment of the rehabilitation protocol was allowed. Patients were asked to wear the knee brace all day long in the first 1.5 months and wear it outdoors in the postoperative 1.5 to 3 months. Three months after the surgery, patients were asked to use the knee brace for exercise training only. The International Knee Documentation Committee (IKDC) questionnaire for knee was completed by each patient through the KNEESUP Care app. Patients filled out the IKDC questionnaires at preoperative day and postoperative 1, 2, 4 and 6 months. In addition to follow the rehabilitation programs in the app, patients met the physical therapists face-to-face once a month. During the meeting, the physical therapists would evaluate the patients’ knee conditions in person, and manual therapies as well as ultrasound therapies were also given to the patients. Six months after the surgery, patients completed both a GNRB arthrometer examination and a Cybex isokinetic dynamometer examination. The thigh circumference was also measured postoperatively.

**Table 1:** The default criterion-based rehabilitation tasks for patients in the present study

Grade	Suggested Post-OP Period	Rehabilitation Tasks
1	1 – 2 weeks	ROM 0 degree Passive stretch for knee extension Quadriceps muscle isometric contraction Straight leg raise Ankle pumping
2	3 – 4 weeks	ROM 0–60 degrees Passive stretch for knee extension Quadriceps muscle isometric contraction Straight leg raise (hip flexion and abduction) Heel slide on wall Lymphatic drainage
3	5 weeks	ROM 0–90 degrees Passive stretch for knee extension in prone position Patella mobilization Heel slide on wall Partial-weight bearing with weight-shifting training Semi-squat and heel-up
4	6—7 weeks	ROM 0–110 degrees Passive stretch for knee extension Patella mobilization Heel slide Full-weight bearing with weight-shifting training Wall squat
5	8—9 weeks	ROM 0–120 degrees Active knee range of motion Lunge for full knee extension training Hamstring contraction exercise Wall squat Stairs up and stairs down
6	10—11 weeks	Full ROM Active knee range of motion Lunge for full knee extension training Plunk and bridging exercise Single leg squat Single leg stands with eyes closed Star exercise Advanced stairs up and stairs down
7	12—13 weeks	Full ROM Squat and single leg squat Kneeling squat Bridging exercise Single leg standing Side steps with thera-band Star exercise
8	14—15 weeks	Full ROM Single leg bridge exercise Side steps with thera-band Single leg standing Stairs up and stairs down with side steps Single leg squat Squat to stand on tiptoe Hop
9	After 16 weeks	Full ROM Lunge with hip external/internal rotation Advanced stairs up and stairs down Advanced single leg squat Single leg crossing cone reach Jump and single leg land Single leg hops in place

*Post-OP* Postoperative, *ROM* Range of motion

### Measurements of GNRB arthrometer and Cybex isokinetic dynamometer

Both the injured and the healthy knees were assessed by GNRB arthrometer and Cybex isokinetic dynamometer, and the dominant sites of limbs were also recorded. The measurement of GNRB arthrometer was in accordance with the previous studies [8, 16, 20, 21], and the data from GNRB testing were automatically collected in the computer with a 0.1 mm accuracy. The side-to-side difference (SSD) of anterior tibial translation under 134 N between the injured knee and the healthy knee was calculated, and the values greater than 3 mm at any follow up were considered as reconstruction failure [16]. The force–displacement curve was created, and slope 2 (S2), defined as the slope of the curve ranging between 100 N and the maximum force, was also acquired (mm/N). The slope of force–displacement was considered as ligamentous elasticity [20]. A Cybex isokinetic dynamometer was used for evaluating the knee flexor (hamstrings muscle) and extensor (quadriceps muscle) strengths. The isokinetic concentric tests were performed at angular velocities of 60°/s for hamstrings muscle and quadriceps muscle. The peak torques of flexor and extensor muscles were recorded, and values were standardized by the patients’ body weights (BW).

### Statistical analysis

Statistical analyses were conducted using SPSS 22.0 (SPSS Inc., Chicago, IL, USA). Descriptive statistics, including means and standard deviations were obtained. As the main purpose of the current study was to compare the postoperative data to preoperative ones, the Wilcoxon matched-pairs signed rank test was used. Statistical significance was set as *p* ≤ 0.05.

## Results

A total of 15 patients (10 males and 5 females) were prospectively enrolled and completed the follow-up for at least 6 months. The demography data of each patient were summarized in Table 2. The knee joint range of motion of each patient were recorded preoperatively and postoperatively, and the mean time of return to full knee extension was 1.5 ± 0.9 months (Table 2). The average time for exercise training through the knee brace was 65 ± 8 min in the first three months, whereas it was 40 ± 8 min in the following 3 months.

**Table 2:** The demographic information and the knee joint range of motion of each patient

Patient No	Gender	Age	Laterality	Preoperative AROM	Preoperative PROM	6 months post-OP AROM	6 months post-OP PROM	Time to reach full knee extension (month)
1	Female	26	Right	0–150	0–155	0–150	0–155	3
2	Female	21	Left	5–70	5–75	0–130	0–130	1
3	Male	22	Right	0–110	0–120	0–121	0–130	1
4	Male	30	Right	0–130	0–142	0–122	0–145	1
5	Male	24	Right	10–140	0–145	0–135	0–140	3
6	Male	42	Right	0–120	0–134	0–115	0–130	1
7	Female	30	Left	0–32	0–46	0–96	0–100	1
8	Male	30	Right	0–128	0–140	0–122	0–132	3
9	Female	41	Left	0–126	0–130	0–120	0–126	1
10	Male	21	Left	0–140	0–143	0–140	0–146	1
11	Female	28	Left	0–120	0–124	0–138	0–142	1
12	Male	31	Left	0–114	0–124	0–120	0–140	1
13	Male	24	Left	0–132	0–146	0–140	0–146	1
14	Male	33	Left	0–120	0–126	0–130	0–130	1
15	Male	21	Left	6–126	2–132	0–130	0–135	3
Average		28 ± 7		1–117	0–125	0–127	0–135	1.5 ± 0.9

*AROM* Active range of motion, *PROM* Passive range of motion, *post-OP* Postoperative

Regarding the results measured by GNRB arthrometer, the SSD of anterior tibial translation under 134 N improved by an average of 1.6 mm at postoperative 6 months (*p* = 0.026, effect size r = 0.421), whereas the SSD of S2 at postoperative 6 months was 4.5 mm/N smaller in average compared to preoperative data (*p* = 0.024, effect size r = 0.407). Both the peak torques of flexor and extensor muscles measured by Cybex isokinetic dynamometer at angular velocities of 60°/s remained untouched at postoperative 6 months compared to preoperative findings. There was also no significant difference in mean thigh circumference between preoperative and 6-month postoperative measurements (Table 3).

**Table 3:** The objective measurements for knee before and 6 months after surgery

	Preoperative	Post-OP 6 months	*P* value	Effect size r
GNRB arthrometer				
SSD of ATT under 134 N	3.4 ± 1.9 mm	1.8 ± 1.6 mm	0.026*	0.421
SSD of S2	8.2 ± 6.5 mm/N	3.7 ± 3.1 mm/N	0.026*	0.407
Cybex isokinetic test				
Peak flexor torque (60°/s)	109 ± 42%BW	115 ± 50%BW	0.733	0.062
Peak extensor torque (60°/s)	170 ± 67%BW	153 ± 83%BW	0.394	0.156
Thigh circumference	52 ± 6 cm	52 ± 5 cm	0.670	0.083

*Post-OP* Postoperative, *SSD* Side-to-side difference, *ATT* Anterior tibial translation, *BW* Body weight

^*^Significant different (*p* < 0.05) between preoperative and postoperative data using Wilcoxon matched-pairs signed rank test

**Fig. 3:**
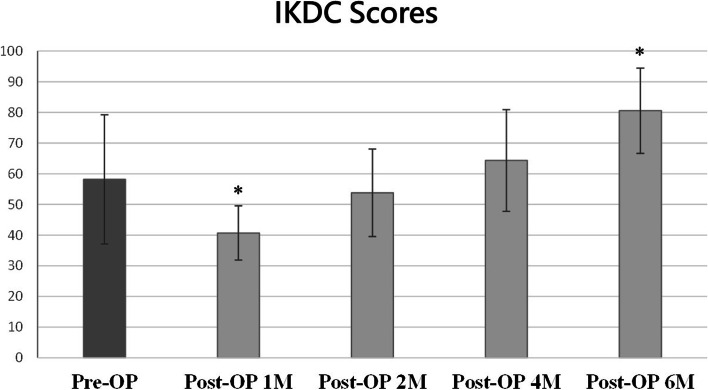
The International Knee Documentation Committee (IKDC) score at preoperative day and postoperative 1, 2, 4 and 6 months. * indicated significantly difference between the preoperative and postoperative scores (*p* < .05)

The IKDC score was 58.2 ± 21 preoperatively, whereas they were 40.7 ± 9, 53.8 ± 14, 64.3 ± 16 and 80.6 ± 14 at postoperative 1, 2, 4, and 6 months, respectively. The IKDC score at postoperative 1 month was significantly smaller than the preoperative score (*p* = 0.011, effect size r = 0.464), whereas the score at postoperative 6 months was significantly greater than the preoperative score (*p* = 0.002, effect size *r* = 0.560) (Fig. 3).

Regarding the responses from users, the major positive feedback from the patients was the clear instructions of exercise in the app. However, some patients felt that it was inconvenient to doing exercise with the knee brace after postoperative 4 months. The most common two questions from the patients to the physical therapist were (1) when could I start knee flexion training? and (2) could I do the exercise if I had pain around the knee?

## Discussion

The major findings of the present study were that the home-based telerehabilitation system was a feasible option after ACL reconstruction as patients maintained their muscle strength and achieve similar or better knee range of motion compared to preoperative measurements. Telerehabilitation is developing, especially during the COVID-19 outbreak [13]. The main treatment goal of postoperative rehabilitation after knee surgery is restoring the range of motion and muscle strength of the knee joint [10]. To achieve better treatment outcomes, newly developed rehabilitation techniques focus more on improving patient motivation and providing more objective monitoring with better feedbacks to patients [3, 10]. In response to the aforementioned purposes, Kim et al. proposed a study protocol that aimed to evaluate the treatment effects of rehabilitation using their wearable device which can measure the range of motion and strength of the knee joint [10]. In the current study, we introduced another novel home-based rehabilitation system, called KNEESUP Compact system, to patients undergoing ACL reconstruction.

The role of knee brace in postoperative rehabilitation is evolving. Traditionally, knee braces were used for rehabilitation since they allow protected motion of the surgically repaired knee [7]. Recently, new knee brace designs are available which potentially improve patient outcomes relative to traditional bracing [7]. In the present study, a home-based rehabilitative knee brace system was introduced to patients after ACL reconstruction. With this rehabilitation system, patients can complete their rehabilitation exercise at home and acquire the real-time feedbacks regarding their rehabilitation status. In other words, the knee brace can have an additional role during the postoperative period. It can not only provide protection force to the knee joint but also act as a rehabilitation training device. Therefore, it could be inferred that the use of the knee brace system for rehabilitation did not negatively affect the knee stability after ACL reconstruction.

The postoperative knee stability is clinically essential. The present study used a validated automated laximeter (GNRB arthrometer) for evaluating the knee joint laxity. The results in the present study indicated that the knee laxity improved significantly after ACL reconstruction. Regarding the degree of postoperative stability, the present study found a mean value of 1.8 mm in SSD of anterior tibial translation under 134 N at postoperative 6 months. In the previous studies [12, 16, 22, 23], values measured by GNRB arthrometer after ACL reconstruction ranged from 1.5 mm to 3.4 mm. Although different studies cannot be compared directly, the results in the present study suggested that the dynamic anterior stability seemed to be adequate when compared with previous studies [12, 16, 22, 23].

Quadriceps strengthening and achieving full knee extension are a major focus of rehabilitation after ACL reconstruction [14]. It is reported that quadriceps atrophy is associated with deficits in performance-based functional tests [11]. The present study used the Cybex isokinetic dynamometer for evaluating the knee muscle strength and found that both the flexion and extension strengths remained at the same level at postoperative 6 months. In other words, using the KNEESUP Compact rehabilitation system prevented muscle wasting during the postoperative period after ACL reconstruction.

Regarding the functional outcomes, the mean IKDC scores collected in the present study improved from 58.2 ± 21 to 80.6 ± 14 at 6 months postoperatively. In the previous studies, the preoperative IKDC scores for patients with ACL tears ranged from 51.6 to 57.1, whereas the postoperative IKDC scores reached 69.5 to 88.9 at 1-year follow up after ACL reconstruction [1, 2, 6, 18]. Although different studies cannot be compared directly, the results of the current study suggested the KNEESUP Compact rehabilitation system to be a reliable option for postoperative rehabilitation after ACL reconstruction surgery as our patients had comparable IKDC scores compared to those in the current literature. Further studies are still required for comparing the treatment effects between the telerehabilitation and the traditional rehabilitation programs.

The KNEESUP Compact rehabilitation system features several advantages. First, the user interface of the mobile app and web portal were user-friendly as orthopedic doctors and physical therapists were involved in the software design. Second, the rehabilitation protocol can be individualized. In recent years, the rigid rehabilitation protocols that center around time intervals following ACL reconstruction have gradually been replaced by criteria-based guidelines [5, 14]. Developed in the same direction, the KNEESUP Compact system allows patients to achieve their individualized criterion-based milestones under the supervision of medical staffs. Third, the rehabilitation exercises at home can be supervised. The demonstration videos and the real-time tracking systems help patients confirm the accuracy of their exercise movements, and the medical staffs can monitor the degree of completion in the rehabilitation programs. Forth, use KNEESUP Compact rehabilitation system can reduce the medical expense and the time spent in the hospital. Traditionally, patients will receive face-to-face rehabilitation courses twice a week during the first 6-month postoperative period in our institute. When KNEESUP Compact rehabilitation system was introduced, the patients only received in-person rehabilitation courses once per month.

The present studies had some limitations. First, it was a prospective case series only, and no control group was provided for comparison. Although some of the results could be compared with the current literature, no implications could be drawn based on the analysis of outcomes towards the validity of the knee brace system, Future studies that compare the treatment effects between home-based rehabilitation system and traditional rehabilitation after ACL reconstruction are still needed. Second, the case number in the present study was relatively small. Third, although the medical expense was supposed to be reduced when the home-based rehabilitation system was applied, there is a lack of cost-effectiveness analysis on this topic. Forth, the follow-up period in the present study was 6 months only.

## Conclusion

Using a home-based rehabilitative knee brace system after ACL reconstruction is a viable option as patients maintained their knee muscle strengths and achieve similar or better knee range of motion six months postoperatively.

## Abbreviations

ACL: Anterior cruciate ligament
MRI: Magnetic resonance imaging
IKDC: International Knee Documentation Committee
SSD: Side-to-side difference
S2: Slope 2
BW: Body weight
